# Higher PRBC transfusion volume and frequency are associated with higher risk of moderate-to-severe bronchopulmonary dysplasia or mortality in extremely preterm infants: A retrospective cohort study

**DOI:** 10.1097/MD.0000000000049489

**Published:** 2026-07-10

**Authors:** Jun Luo, Liyuan Liu, Yan Zhang, Yangying Ou, Hung Chih Lin, Wenbin Li

**Affiliations:** a Department of Neonatology, Shenzhen Baoan Women’s and Children’s Hospital, Shenzhen, Guangdong, China; b Department of Neonatology, Shenzhen Longhua District Maternity & Child Healthcare Hospital, Shenzhen, Guangdong, China; c Department of Neonatology, Sun Yat sen Memorial Hospital, Sun Yat sen University, Guangzhou, Guangdong, China; d Department of Pediatrics, China Medical University Children’s Hospital, Taichung, Taiwan; e Department of Pediatrics, Asia University Hospital, Asia University, Taichung, Taiwan; f Department of Pediatrics, Tongji Hospital, Tongji Medical College, Huazhong University of Science and Technology, Wuhan, Hubei, China.

**Keywords:** bronchopulmonary dysplasia, extremely preterm infants, mortality, PRBC transfusion

## Abstract

Several studies have reported inconsistent findings regarding the impact of packed red blood cell (PRBC) transfusions in extremely preterm infants (EPIs). Using a retrospective cohort study design, we investigated the relationship between different volumes and frequencies of PRBC transfusions and the morbidity and mortality in this vulnerable population. During the 3-year research period, our study encompassed 146 infants, ultimately enrolling 101 after a comprehensive screening process. Based on the medium volume of PRBC transfusions, 50 EPIs were classified into a higher-volume PRBC transfusion group (higher PRBC volume transfusion [HVT], > 45.5ml) and 51 infants were in the lower volume PRBC transfusion group (lower PRBC volume transfusion [LVT], < 45.5ml). Complications included intraventricular hemorrhage, hemodynamically significant patent ductus arteriosus, necrotizing enterocolitis, retinopathy of prematurity, bronchopulmonary dysplasia (BPD), and mortality were compared between groups. Statistical analysis was carried out using IBM SPSS Statistics. In the HVT group, 5 infants did not develop BPD while 45 did; among these, 28 (56%) developed mild BPD, 11 moderate, and 6 severe. Critically, no patients in the LVT group developed severe BPD. The study showed HVT infants had differences in characteristics and outcomes compared to those with LVT. There were no significant differences in the rates of intraventricular hemorrhage, hemodynamically significant patent ductus arteriosus, necrotizing enterocolitis, and retinopathy of prematurity (more than II level) between the 2 groups. After adjustment for potential confounding risk factors, higher PRBC transfusion volume was associated with elevated odds of moderate-to-severe BPD or mortality (adjusted odds ratio 2.839, 95% confidence interval [CI]: 1.006–8.014, *P* = .049). A significant positive correlation was identified between higher volumes of PRBC transfusions (*R* = 0.371, *P* < .001) and the frequency of transfusions (*R* = 0.320, *P* = .001) with moderate-to-severe BPD or mortality. Additionally, both volume and frequency of transfusion demonstrated moderate predictive accuracy for moderate-to-severe BPD or mortality in EPIs, with cutoff 45.5 ml and 4 times, with areas under the curve of 0.69 (95% CI: 0.58–0.79, *P* < .05) and 0.7 (95% CI: 0.59–0.80, *P* < .05), respectively. Our findings indicate that both volume and frequency of transfusions were associated with an increased risk of moderate-to-severe BPD or mortality in EPIs. Clinicians should exercise increased caution regarding PRBC transfusions, even when adhering to restrictive transfusion criteria.

## 1. Introduction

In 2020, preterm infants accounted for 9.9% of newborns,^[1]^ among them, 4% were extremely preterm infants (EPIs) with a gestational age (GA) of < 28 weeks.^[2]^ Although management and outcomes of EPIs have improved, various complications for these premature infants still exist and have been increasing annually^.[3–5]^

EPIs are at risk for complications that require extensive blood sampling and subsequent packed red blood cell (PRBC) transfusions.^[6]^ However, it is important to note that blood sampling and transfusion-related procedures may lead to pain, decreased oxygen saturation, hypoxic episodes, and a drop in blood pressure. Evidence suggests that transfusions have been associated with mortality,^[7]^ severe intraventricular hemorrhage (IVH),^[8]^ necrotizing enterocolitis (NEC),^[9]^ severe retinopathy of prematurity (ROP),^[10]^ and bronchopulmonary dysplasia (BPD).^[11]^ Current evidence remains conflicting regarding the complications and mortality associated with blood transfusions in very preterm infants.^[12–14]^ Nevertheless, uncertainties persist regarding the potential risks of PRBC transfusion exposure in EPIs. Heterogeneity may reflect variations in demographic characteristics such as GA and birth weight, criteria for blood sampling, and the frequency or amount of PRBC transfusion across different neonatal intensive care unit centers.

A key mechanism for oxidative damage from these transfusion procedures is the release of heme iron following the breakdown of erythrocytes. Serum iron, non-transferrin-bound iron (NTBI), and monocyte chemoattractant protein levels were significantly elevated following the transfusion of PRBCs.^[15]^ Another study indicated that red blood cell (RBC) transfusions in very low birth weight infants were associated with heightened markers of hemolysis and the inflammatory chemokine monocyte chemoattractant protein-1. The duration of RBC storage was correlated with increases in NTBI levels following transfusion^.[16]^

According to the above studies, we hypothesized that EPIs receiving higher transfusion volume and frequency of PRBC transfusions would generate more free radicals and inflammatory mediators, thereby increasing the risk of morbidity and mortality. This study aims to further investigate the relationship between the volume and frequency of PRBC transfusions and morbidity/mortality in EPIs.

## 2. Methods

This study included 101 EPIs (< 28 weeks’ GA) admitted to Shenzhen Bao’an Women’s and Children’s Hospital affiliated with Jinan University and Longhua District Women’s and Children’s Hospital between January 2020 and December 2022. Inclusion criteria were admission within 24 hours after birth and hospital stays of ≥ 14 days. Among the 101 EPIs enrolled, the median (P25, P75) volume of PRBC transfusion was 45.5ml (36.5, 60) during hospital stay. These EPIs were consecutively divided into 2 groups based on the median PRBC transfusion of 45.5ml: 50/101 (49.5%) as higher PRBC volume transfusion (HVT) and 51/101 (50.5%) as lower PRBC volume transfusion (LVT). Exclusion criteria included parental refusal for consent, transfer or discharge within 2 weeks, family history of anemia, fetal-to-maternal blood transfusion, fetal-to-fetal blood transfusion, neonatal hemorrhagic diseases, major congenital anomalies, or incomplete hospitalization data. All infants underwent delayed cord clamping (≥ 60 seconds). The study protocol has obtained approval from the local Ethics Committees, with approval numbers LLSC-2023-03-10-02-KS and SRE-PCFR/2025038, respectively (See Table S1, Supplemental Digital Content 1). This approval includes waivers of informed consent from family members for both participating institutions.

We retrospectively analyzed maternal and infant clinical records, including maternal and obstetrical information, demographics, neonatal morbidity, and mortality recorded during hospitalization. GA was assessed by the last menstrual period, prenatal ultrasounds, and postnatal physical examinations. Chorioamnionitis was defined as an elevated maternal temperature (≥ 38°C) accompanied by 2 or more of the following symptoms or signs: increased pulse (> 100 beats/min), increased fetal heart rate (> 160 beats/min), tenderness of the uterine floor, foul-smelling amniotic fluid, and elevated white blood cell levels (> 15 × 10^9^/L or nuclear shift to the left)^.[17]^ Prenatal steroid use was defined as maternal receipt of glucocorticoids before delivery. Neonatal sepsis was confirmed by clinical manifestations plus positive blood culture results.^[18]^ Early-onset sepsis (EOS) and late-onset sepsis (LOS) were considered if diagnosed before and after 72 hours of life.

The severity of EPIs at admission was assessed using the Score for Neonatal Acute Physiology Ⅱ or the Score for Neonatal Acute Physiology, Perinatal Extension, Version Ⅱ; detailed data on these scores are presented in the supplementary table. BPD was defined based on the criteria established by the National Institute of Health,^[19]^ where infants who required positive pressure support or at least 30% supplemental oxygen at 36 weeks postmenstrual age or prior to discharge (whichever occurred first) were classified as having severe BPD. Those requiring 21 to 30% supplemental oxygen were categorized as having moderate BPD. Staging for ROP followed International Classification guidelines.^[20]^ NEC was defined as stage 2 or higher based on Bell criteria.^[21]^ Small for GA was defined as birth weight below the 10th percentile based on Fenton Growth Chart standards.^[22]^ IVH was classified according to Papile criteria.^[23]^ Extrauterine growth restriction (EUGR) was defined as weight at discharge below the 10th percentile (*z*-score ≤ 1.28).^[24]^ Hemodynamically significant patent ductus arteriosus (hsPDA) was defined as signs of PDA plus the presence of cardiomegaly and pulmonary edema on chest radiography, as well as at least 1 of the following: internal ductal diameter ≥ 1.5 mm, left-atrium-to-aortic-root ratio > 1.6, unrestricted pulsatile trans-ductal flow, or reverse or absent diastolic flow in the descending aorta.^[25]^

The criteria for PRBC transfusion for EPIs were as follows: In critical scenarios, for infants aged ≤ 7 days, hemoglobin (Hb) levels should be < 115 g/L; for those aged 8 to 21 days, Hb < 100 g/L; and for infants older than 21 days, Hb < 90 g/L. In noncritical situations, the thresholds are: for infants aged ≤ 7 days, Hb < 95 g/L; for those aged 8 to 21 days, Hb < 80 g/L; and for infants older than 21 days, Hb < 70 g/L.^[26]^ A condition was deemed critical if any of the following conditions were met: requiring invasive mechanical ventilation; necessitating continuous positive airway pressure with a fraction of inspiration oxygen (FiO_2_) exceeding 0.25 for more than 12 hours per day; having a patent ductus arteriosus that required intervention; experiencing more than 6 episodes of apnea that necessitated stimulation for resolution within a 24-hour period, or more than 4 hypoxic episodes (pulse oxygen saturation [SpO_2_] < 60%) despite the administration of methylxanthines and continuous positive airway pressure; or suffering from circulatory failure due to sepsis or NEC, which required cardiac and/or vascular support therapy^.[26]^

Categorical variables were shown as proportions n/N (%) and compared with the Chi-square (*χ*^2^) test. Continuous variables were presented as mean ± standard deviation or median and interquartile range (P25, P75), and compared using Student *t* test or a nonparametric test as appropriate. Two-tailed *P* values < .05 were deemed statistically significant. Additionally, logistic regression was performed for multivariate analysis to evaluate predictors of neonatal morbidities and mortality. Pearson correlation coefficient was used to investigate the relationship between moderate-to-severe BPD or mortality and GA, invasive mechanical ventilation, EOS, LOS, transfusion (ml), and transfusion times. Statistical analyses were carried out using IBM SPSS Statistics (version 20).

## 3. Results

### 3.1. Clinical characteristics of EPIs with HVT or LVT

**Figure 1.**
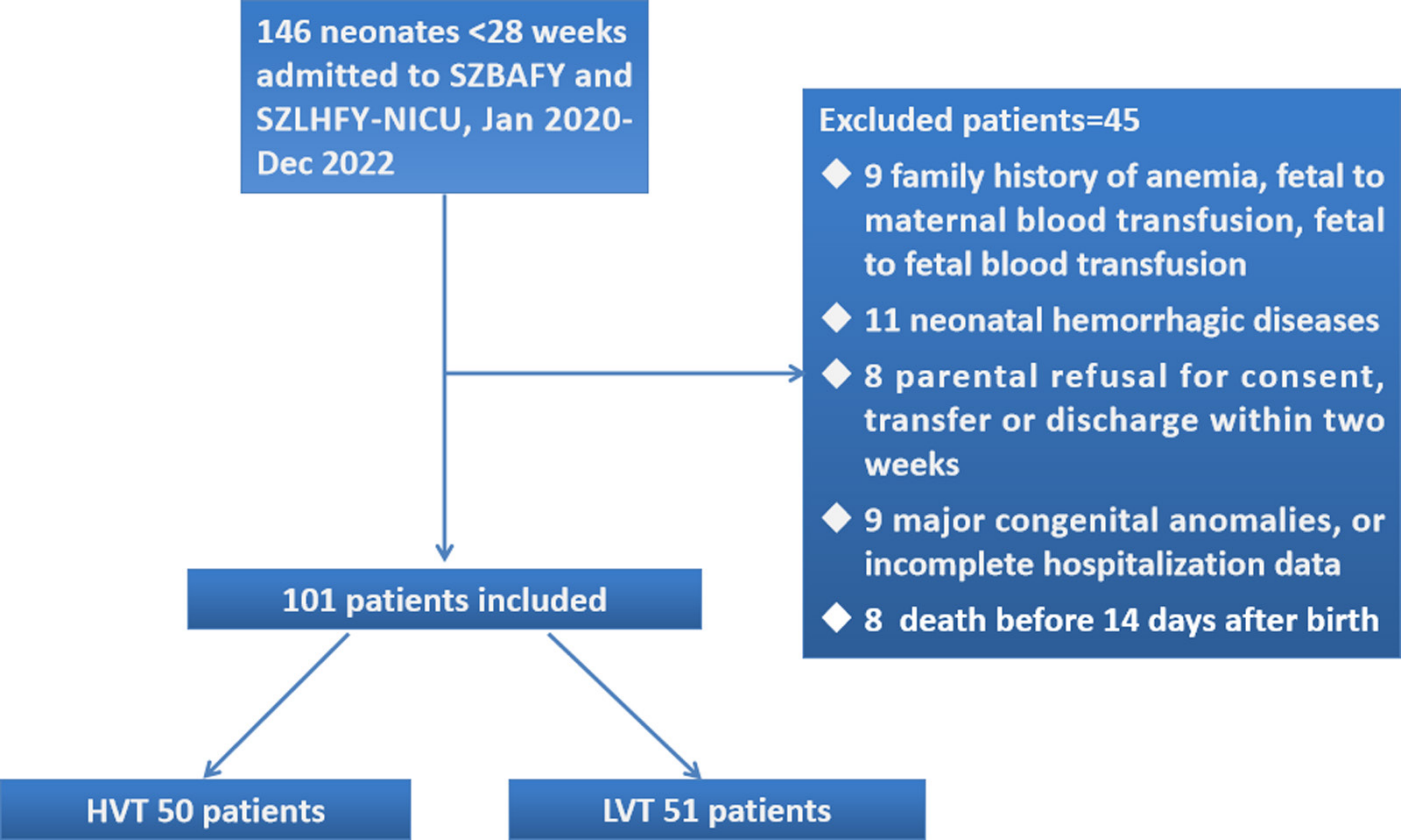
Study flow diagram. HVT = higher PRBC volume transfusion, LVT = lower PRBC volume transfusion, SZBAFY = Shenzhen Bao’an Women’s and Children’s Hospital affiliated with Jinan University, SZLHFY = Longhua District Women’s and Children’s Hospital.

During the 3-year research period, our study assessed 146 infants. Following a thorough screening process, we included 101 patients, as detailed in Figure 1. We then studied these 101 infants born at < 28 weeks of gestation, including 81 infants classified as extremely low birth weight, weighing < 1000 grams. Out of these, 14 infants (14%) did not develop BPD, while 87 did; of those, 60 (60%) had mild BPD, 21 (20%) moderate BPD, and 6 (6%) severe BPD.

Hb levels differed significantly between the HVT and LVT groups during specific postnatal periods. As detailed in Table 1, factors such as the prevalence of delivery via cesarean section (C-section), birth asphyxia, EOS, duration of invasive mechanical ventilation, PRBC transfusion volumes (ml), frequency of PRBC transfusion, GA, and birth weight were significantly higher in the HVT group compared to the LVT group. There was no significant difference in Hb levels within the first 24 hours between the HVT and LVT groups in EPIs. Additionally, no significant differences were identified between the groups regarding gender, chorioamnionitis, mode of pregnancy, multiple births, antepartum hemorrhage, antenatal glucocorticoids, LOS, maternal Hb levels, and maternal hematocrit levels. The incidence of preeclampsia in both groups was relatively low; thus, it was omitted due to low incidence. When assessed by the Score for Neonatal Acute Physiology Ⅱ or the Score for Neonatal Acute Physiology, Perinatal Extension, Version Ⅱ, the admission severity of EPIs showed no significant difference between groups (Table S1, Supplemental Digital Content 1).

**Table 1:** Clinical characteristics of EPIs with HVT compared to LVT.

	HVT group (n = 50)	LVT group (n = 51)	*P* †
Male, n (%)	34	28	.176
Chorioamnionitis, n (%)	7	3	.302
Cesarean delivery, n (%)	15	28	.011*
Multiple pregnancies, n (%)	17	25	.126
Placental abruption, n (%)	4	2	.436
Antenatal glucocorticoids(≥4 times), n (%)	27	32	.373
Birth asphyxia, n (%)	19	10	.041*
Early-onset sepsis, n (%)	35	17	< .001*
Late-onset sepsis, n (%)	6	3	.318
Maternal Hb level before delivery (g/L), mean ± SD	112.14 ± 17.85	106.41 ± 15.40	.087
Maternal HCT level before delivery, mean ± SD	0.34 ± 0.05	0.32 ± 0.04	.187
Invasive mechanical ventilation (hours), median (IQR)	273.84 (112.44–584.77)	66.80 (25.00–113.25)	< .001*
PRBC transfusion volumes (ml/kg), median (IQR)	70.59 (55.95–112.33)	38.58 (32.22–47.06)	< .001*
PRBC transfusion volumes (ml), median (IQR)	60.00 (50.00–81.25)	37.00 (30.00–40.00)	< .001*
Frequency of PRBC transfusion^b^, median (IQR)	4 (2–6)	2 (1–2)	< .001*
Gestational age (week)‡, median (IQR)	26.15 (25.00–27.00)	27.14 (26.29–27.57)	< .001*
Birth weight (grams), mean ± SD	829.10 ± 167.25	942.67 ± 177.57	.001*
Hb level within 24 hours (g/L), mean ± SD	141.80 ± 26.23	147.96 ± 22.17	.205

EPI = extremely preterm infants, Hb = hemoglobin, HCT = hematocrit, HVT = higher PRBC volume transfusion, IQR = interquartile range, LVT = lower PRBC volume transfusion, n = number of participants, PRBC = packed red blood cells, SD = standard devitation.

† *P* values are from chi-square or Fisher exact tests.

‡ Indicates that it has been tested as non-normal by SK.

* *P* < .05.

Given the inherent baseline imbalances in GA, birth weight, invasive ventilation duration, birth asphyxia, and EOS (all well-established independent risk factors for BPD and neonatal mortality) these variables were deliberately selected and fully adjusted for in the primary multivariate regression models to mitigate confounding bias.

### 3.2. The Impact of HVT on morbidity and mortality

In the HVT group, 5 infants did not develop BPD, while 45 did; among these, 28 (56%) developed mild BPD, 11 moderate, and 6 severe. Notably, no patients in the LVT group developed severe BPD. Rates of patients diagnosed with IVH (34.00% vs 11.76%, odds ratio [OR]: 3.864, 95% confidence interval [CI]: 1.375–10.860, *P* = .008), EUGR (26.00% vs 7.84%, OR: 4.128, 95% CI: 1.243–13.715, *P* = .015), and moderate-to-severe BPD or mortality (46.00% vs 19.61%, OR: 3.493, 95% CI: 1.438–8.481, *P* < .001) in the HVT cohort were significantly higher compared to the LVT cohort. We found no differences in IVH and EUGR after adjusting for potential risk factors, nor in the morbidity and mortality associated with BPD, hemodynamically significant PDA, ROP (> level II), LOS, and NEC between the 2 groups (Table 2). However, significant disparities remained in the occurrence of moderate-to-severe BPD or mortality (adjusted OR: 2.839, 95% CI: 1.006–8.014, *P* = .049) after adjusting for GA, birth weight, birth asphyxia, delivery mode, and EOS between the 2 groups.

**Table 2:** The impact of higher PRBC volume transfusion on morbidity and mortality.

	HVT group (n = 50)	LVT group (n = 51)	Crude OR(95%CI)	Adjusted OR† (95%CI)
BPD	45 (90.00%)	42 (82.35%)	1.929 (0.598–6.222)	-
hsPDA	27 (54.00%)	21 (41.18%)	1.677 (0.763–3.683)	-
ROP (more than II level)	26 (52.00%)	25 (49.02%)	1.127 (0.516–2.459)	-
NEC	7 (14.00%)	2 (3.92%)	3.988 (0.786–20.234)	-
Late-onset sepsis	6 (12.00%)	3 (5.88%)	2.182 (0.514–9.255)	-
IVH	17 (34.00%)	6 (11.76%)	3.864 (1.375–10.860)*	2.014 (0.613–6.620)
EUGR	13 (26.00%)	4 (7.84%)	4.128 (1.243–13.715)*	3.441 (0.848–13.962)
Moderate-to-severe BPD or mortality	23 (46.00%)	10 (19.61%)	3.493 (1.438–8.481)*	2.839 (1.006–8.014)*

BPD = bronchopulmonary dysplasia, CI = confidence interval, EOS = early-onset sepsis, EUGR = extrauterine growth restriction, hsPDA = hemodynamically significant patent ductus arteriosus, HVT = higher PRBC volume transfusion, IVH = intraventricular hemorrhage, LVT = lower PRBC volume transfusion, NEC = necrotizing enterocolitis, n = number of participants, OR = odds ratio, ROP = retinopathy of prematurity.

† adjusted for gestational age, birth weight, birth asphyxia, delivery mode and EOS.

* *P* < .05.

### 3.3. Factors associated with moderate to severe BPD or mortality

As illustrated in Table 3, the Pearson correlation coefficient was used to assess the relationship between moderate-to-severe BPD or mortality and variables such as GA, invasive mechanical ventilation, EOS, LOS, PRBC transfusion volumes (ml), and frequency of transfusions. The findings indicated a significant positive correlation between PRBC transfusion volumes (*R* = 0.371, *P* < .001), frequency of transfusions (*R* = 0.320, *P* = .001), prolonged invasive mechanical ventilation (*R* = 0.328, *P* = .001), and LOS (*R* = 0.301, *P* = .002) with moderate-to-severe BPD or mortality. Conversely, GA exhibited a significant negative correlation with moderate-to-severe BPD or mortality (*R* = −0.244, *P* = .014), while EOS showed no correlation with moderate-to-severe BPD or mortality (*R* = 0.085, *P* = .399).

**Table 3:** Correlation between moderate-to-severe BPD or mortality with gestational age, invasive mechanical ventilation, EOS, LOS, PRBC transfusion volumes (ml) and transfusion times in extremely preterm infants.

Variables	*r*	*P*
Gestational age	−0.244	.014*
Invasive Mechanical Ventilation	0.328	.001*
EOS	0.085	.399
LOS	0.301	.002*
PRBC transfusion volumes (ml)	0.371	< .001*
Frequency of PRBC transfusion	0.320	.001*

BPD = bronchopulmonary dysplasia, EOS = early-onset sepsis, LOS = late-onset sepsis, PRBC = packed red blood cell.

* *P* < .05.

### 3.4. Assessment of the impact of the amounts of PRBC transfusion on moderate to severe BPD or mortality

The optimal thresholds for PRBC transfusion volume and frequency, as determined by the receiver operating characteristic curve, were 45.5 ml and 4 times, respectively, yielding an area under the curve (AUC) of 0.69 and 0.7 (*P* < .05). The sensitivity and specificity for PRBC transfusion volume were 70% and 60%, while for the frequency of PRBC transfusion in EPIs, the sensitivity was 58% and the specificity was 80%.

**Figure 2.**
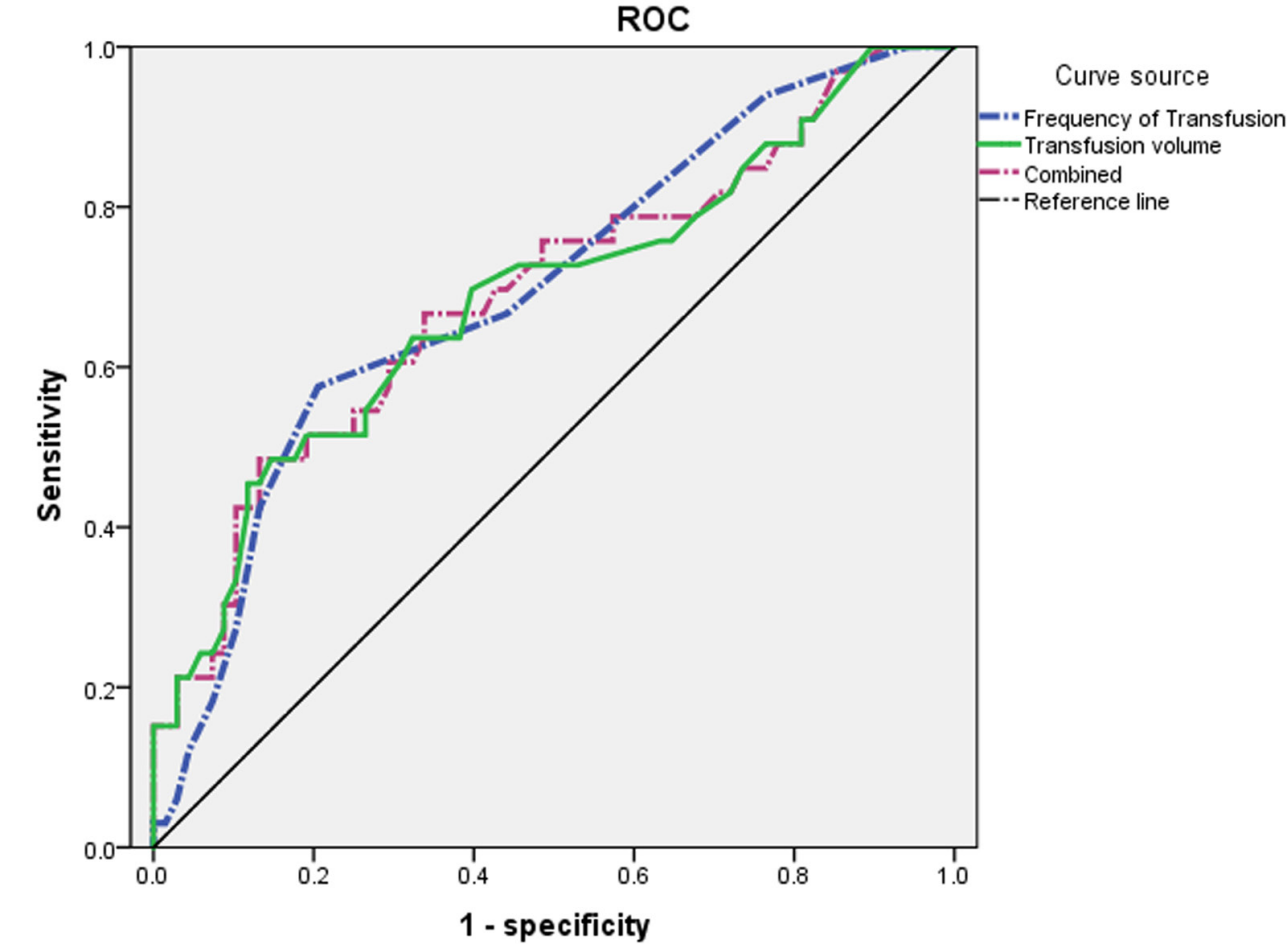
ROC curve illustrating the predictive value of PRBC transfusion volume, frequency of PRBC transfusion, and their combination concerning the incidence of moderate-to-severe BPD or mortality. The green and blue lines represent the ROC curves for PRBC transfusion volume and frequency, predicting the occurrence of moderate-to-severe BPD or mortality, with AUCs of 0.69 (95% CI: 0.58–0.79, *P* < .05) and 0.7 (95% CI: 0.59–0.80, *P* < .05), and cutoff levels of 45.5 ml (sensitivity 70%, specificity 60%) and 4 times (sensitivity 58%, specificity 80%) in EPIs, respectively. The combined analysis (purple line) of these 2 markers did not improve the diagnostic accuracy for moderate-to-severe BPD or mortality in this patient cohort, maintaining an AUC of 0.69 (95% CI: 0.58–0.79, *P* < .05), with sensitivity at 67% and specificity at 62%. The black line indicates the reference line. AUC = area under the curve, BPD = bronchopulmonary dysplasia, CI = confidence interval, EPI = extremely preterm infants, PRBC = packed red blood cell, ROC = receiver operating characteristic.

The combination of these 2 markers did not enhance the diagnostic accuracy for moderate-to-severe BPD or mortality in these patients, with an AUC of 0.69, sensitivity of 67%, and specificity of 62%. The receiver operating characteristic curve indicated a moderate predictive capability regarding the volume of PRBC transfusion and frequency in relation to moderate-to-severe BPD or mortality (see Fig 2).

## 4. Discussion

Although prior studies have explored the impact of PRBC transfusions in very preterm infants (< 32 weeks), data remain limited due to a lack of comprehensive research on the morbidity and mortality associated with PRBC transfusion in EPIs throughout hospitalization. In this multicenter retrospective cohort study, we found a correlation between the volume and frequency of PRBC transfusions and increased rates of moderate-to-severe BPD or mortality in EPIs.

A recent systematic review and meta-analysis indicates that there was no direct association between PRBC transfusion and BPD, consistent with our results.^[13]^ In contrast, a retrospective study involving 679 ELBW infants from 2018 to 2022 reported an association between PRBC transfusions and both the incidence and severity of BPD. Additionally, this study indicated that adherence to current restrictive transfusion guidelines could lead to a modest yet significant decrease in BPD rates.^[11]^ Another study found that a higher number and volume of transfusions were associated with BPD in very preterm infants, with a PRBC transfusion volume ≥ 42 ml/kg identified as a statistically significant predictor for BPD development at a postmenstrual age of 36 weeks.^[27]^ Notably, our study indicates that while an increase in PRBC transfusions may not directly elevate the incidence of BPD, higher PRBC transfusions may raise the combined occurrence of moderate-to-severe BPD or mortality. Variations in research subjects, BPD incidence, and clinical characteristics could explain divergent results observed in our study compared to the findings of Bolat et al.^[27]^

The potential role of PRBC transfusion in the development of ROP, IVH, NEC, and EUGR remained controversial. Wang et al^[28]^ reported that PRBC transfusion in very preterm infants significantly raises the risk of ROP, with the risk escalating alongside the volume of transfusions. We compared multiple with fewer PRBC transfusions, whereas the aforementioned study contrasts PRBC transfusions with no transfusions at all, leading to differing results regarding ROP. In most very preterm infants, IVH was already present at the time of the initial PRBC transfusion,^[29]^ which precludes causal inference of the relationship between PRBC transfusion and IVH. Our study did not specify the timing of initial PRBC transfusions, preventing a direct comparison of incidence with other studies.

A secondary analysis of a randomized clinical trial by Salas et al indicated that exposure to RBC transfusions was not temporally linked to an increased risk of NEC during the 72-hour posttransfusion hazard periods among extremely low birth weight infants within the Hb ranges observed in the TOP (Transfusion of Premature) trial.^[14]^ Our findings corroborate this, suggesting that a higher number of PRBC transfusions in EPIs may not correlate with NEC. EUGR remains a significant challenge in EPIs, indicating severe early-life nutritional deficiencies and an increased risk of growth impairment in childhood, along with potential long-term health complications. Marques et al identified anemia requiring RBC transfusions as an independent risk factor for EUGR.^[30]^ The differing study populations may partially account for the variations compared to our results. Therefore, prospective studies with substantially larger sample sizes are essential to clarify the associations between PRBC transfusion with ROP, IVH, NEC, and EUGR.

While we observed a statistically significant difference in EOS incidence between HVT vs LVT groups (*P* < .001), multivariate analysis (Table 3) did not demonstrate an independent association between EOS and the composite outcome of moderate-to-severe BPD or mortality. This finding may reflect the complexity of disease progression in critically ill preterm neonates. Although those requiring intensive monitoring often undergo repeated blood sampling (potentially necessitating higher transfusion volumes) our data suggest that the increased transfusion burden during the early neonatal period does not appear to significantly impact the subsequent development of severe BPD. These results align with emerging evidence that severe BPD pathogenesis is multifactorial, where factors such as oxidative stress, ventilator-induced lung injury, and postnatal growth restriction may play more dominant roles than transfusion-related effects alone.^[31]^

### 4.1. Strength and limitations of the study

The small sample size is the limitation of this retrospective study. A post hoc power analysis confirmed that the 101-sample size provides a statistical power of > 80% (*α* = 0.05), which is sufficient to detect the core association between the 2 groups. Expanding the sample size is needed to further prove the impact of PRBC transfusion on these EPIs on moderate-to-severe BPD, IVH, and EUGR. Consequently, our findings may be most applicable to similar tertiary neonatal intensive care unit settings with comparable patient demographics and clinical practices, to avoid overgeneralization to broader populations. Second, potential unmeasured confounding factors, such as minor comorbidities not documented in electronic medical records, could introduce residual bias to our analysis. Specifically, respiratory support modes and nutritional support protocols (key clinical variables in EPI management) may interact with transfusion burden to modulate BPD risk. High-frequency oscillatory ventilation, commonly used for EPIs with severe respiratory distress, imposes mechanical stress on immature lung tissue that may synergize with transfusion-related oxidative stress, exacerbating alveolar epithelial injury and subsequent BPD development. Furthermore, the duration of RBC storage was associated with elevated levels of NTBI following transfusion,^[16]^ although we lacked data on this aspect. Importantly, residual confounding by indication remains a major concern: clinically sicker infants tend to receive more frequent and HVT while also possessing a higher baseline risk of BPD and mortality. Lastly, the retrospective design might be another limitation of this study. However, due to multiple ethical considerations, it is nearly impossible to use a prospective randomized controlled trial to investigate the impact of PRBC transfusion on the development and progression of BPD in EPIs.

The study’s strength was that this study focused on extremely immature infants and the utilization of detailed transfusion data from blood transfusions in this specific patient population. By investigating the risk factors linked to PRBC transfusions in EPIs and their impact on mortality and morbidity during hospitalization, the study provides clinically relevant insights for the prevention of common complications in these vulnerable patients. We recognize that future prospective research could further elucidate the underlying mechanisms. Such studies should incorporate enhanced data collection protocols for previously unmeasured confounders (e.g., detailed nursing interventions, serial inflammatory factor levels including interleukin-6 and tumor necrosis factor-α), optimize statistical models with refined parameter adjustment, and integrate relevant biomarkers (e.g., NTBI, antioxidant enzyme activity). Alternative study designs (such as multicenter prospective cohorts with prespecified variable collection or propensity score matching to balance baseline differences) offer feasible approaches to validate the stability of our conclusions while adhering to ethical constraints.

In conclusion, PRBC transfusions exceeding 45.5 ml and occurring more than 4 times had AUC values of 0.69 and 0.7 (*P* < .05). We found that both the volume and frequency of transfusions predicted moderate-to-severe BPD or mortality in EPIs. Clinicians should cautiously limit PRBC transfusions, even when adhering to restrictive transfusion criteria.

## Acknowledgments

This study was supported by grants from China Medical University Hospital (DMR-107-050) and Asia University Hospital (ASIA-111–51004).

## Author contributions

**Conceptualization:** Wenbin Li.

**Data curation:** Jun Luo, Yan Zhang, Hung Chih Lin..

**Formal analysis:** Liyuan Liu, Hung Chih Lin.

**Investigation:** Liyuan Liu, Yan Zhang, Yangying Ou.

**Methodology:** Jun Luo, Yan Zhang, Hung Chih Lin.

**Project administration:** Yangying Ou.

**Resources:** Wenbin Li.

**Software:** Jun Luo, Liyuan Liu, Yangying Ou, Hung Chih Lin.

**Supervision:** Liyuan Liu, Wenbin Li.

**Validation:** Yangying Ou.

**Writing** – **original draft:** Jun Luo, Liyuan Liu, Yangying Ou, Wenbin Li.

**Writing** – **review & editing:** Jun Luo, Hung Chih Lin.


