# Supplementary material for: Higher PRBC transfusion volume and frequency are associated with higher risk of moderate-to-severe bronchopulmonary dysplasia or mortality in extremely preterm infants: A retrospective cohort study
Source: Medicine (Baltimore). 2026 Jul 10;105(28):e49489. doi: 10.1097/MD.0000000000049489 (PMC13363128; doi:10.1097/MD.0000000000049489)
Supplement: Supplementary file 1 [file medi-105-e49489-s001.docx]

**Supplementary Table: Critical illness scores and timing of first packed red blood cell (PRBC) transfusion [median (range)] in extremely preterm infants (EPIs) stratified by PRBC transfusion volume.**

|  | HVT group  （n=50） | LVT group（n=51） | *P* |
| --- | --- | --- | --- |
| SNAP-Ⅱ, median (range) | 6.50（0~40.00） | 5.00（0~36.00） | 0.669 |
| SNAPPE-Ⅱ,  median (range) | 17.5（10.00~64.00） | 10（0~63.00） | 0.082 |
| CRIB-Ⅱ,  median (range) | 11.50（7.00~13.00） | 8.50（7.00~ 12.00） | 0.056 |
| Timing of first transfusion (d),  median (range) | 1.76（0.27~31.78） | 2.02（0~24.43） | 0.480 |

This table presents the distribution of three validated neonatal critical illness scores (SNAP-II, SNAPPE-II, CRIB-II) and the timing of first PRBC transfusion in 101 EPIs, grouped by PRBC transfusion volume (HVT: n=50; LVT: n=51). All data are expressed as median (range), with P values indicating the statistical difference between the HVT and LVT groups (P> 0.05 for all indices, suggesting no significant differences in baseline critical illness severity or timing of first transfusion between the two groups). HVT = higher PRBC volume transfusion group; LVT = lower PRBC volume transfusion group; SNAP-II = Score for Neonatal Acute Physiology II; SNAPPE-II = Score for Neonatal Acute Physiology, Perinatal Extension, Version II; CRIB-II = Clinical Risk Index for Babies II; d = day.
